# Potential impact of L-threonine and encapsulated butyric acid (ButiPEARL) on growth performance, serum biomarkers, intestinal histomorphometry and economic indices of Nile tilapia fingerlings

**DOI:** 10.1186/s12917-025-04590-6

**Published:** 2025-03-31

**Authors:** Hadeer A. Youssuf, Ahmed Shehab, Amira M. Abd-El Hamed, Walaa S. Raslan, Yasmeen Magdy, Mona Gamel Mohamed, Amgad Kadah, Aya F. Matter

**Affiliations:** 1 Aquatic Animal Medicine Department, Faculty of Veterinary Medicine, Benha University, Moshtohor, Toukh, 13736 Qalyubia Egypt; 2 Department of Nutrition and Clinical Nutrition, Faculty of Veterinary Medicine, Benha University, Moshtohor, Toukh, 13736 Egypt; 3 Economics and Farm Management, Department of Animal Wealth Development, Faculty of Veterinary Medicine, Benha University, Moshtohor, Toukh, 13736 Qalyubia Egypt; 4 Physiology Department, Faculty of Veterinary Medicine, Benha University, Moshtohor, Toukh, 13736 Qalyubia Egypt; 5 Anatomy and Embryology Department Faculty of Veterinary Medicine, Benha University, Moshtohor, Toukh, 13736 Qalyubia Egypt; 6 Egyptian Drug Authorities, Dokki, Giza Egypt

**Keywords:** Encapsulated butyric acid, Growth performance, Immunity, L-threonine, *Oreochromis niloticus*, Profitability

## Abstract

**Background:**

Numerous immunostimulants have been incorporated as additives in aquaculture feed due to their potential to improve growth and improve immune function. The present study investigated the effects of dietary L-threonine and an encapsulated butyric acid (ButiPEARL) on several parameters related to Nile tilapia, *Oreochromis niloticus*, specifically focusing on growth performance and immune response.

**Materials and methods:**

A total of 486 Nile tilapia fingerlings were divided into six experimental fish groups (*n* = 81). Each replicate consisted of 27 fish that were used, weighing approximately 15 ± 0.5 (g) and measuring 7 ± 0.5 (cm). The fish in the control group (G1) were fed a basal diet, while the fish in the other groups were fed experimental diets that included varying concentrations of butyric acid and L-threonine per kilogram of diet, as the second group (G2) fed on a basal diet supplemented with butyric acid 0.3 g/kg diet, the third group (G3) fed on a basal diet supplemented with L-threonine 0.24 g/kg diet, the fourth group (G4) fed on a basal diet supplemented with butyric 0.3 g/kg + L-threonine 0.24 g/kg diet, the fifth group (G5) fed on a basal diet supplemented with L-threonine 0.48 g/kg diet, and the sixth group (G6) fed on a basal diet supplemented with butyric 0.3 g/kg + L-threonine 0.48 g/kg. The feeding was done twice a day for 42 days, with the amount of food given being 5% of the fish's body weight. The water parameters were maintained at suitable levels.

**Results:**

The supplementation of these feed additives, particularly the group of fish fed a diet containing 0.3 g/kg of encapsulated butyric acid (ButiPEARL) mixed with 0.48 g/kg of L-threonine, exhibited the highest values throughout the experiment. This supplementation significantly enhanced growth performance indicators (*P* < 0.05), such as length gain, weight gain, weight gain rate (WGR), specific growth rate (SGR), and feed conversion ratio (FCR). Furthermore, it positively influenced immunological parameters (IgM, albumin, total protein in serum, and nitrous oxide in liver tissue), liver enzymes (ALT, AST in serum), antioxidants (superoxide dismutase and catalase) and malondialdehyde in liver), biochemical assays (plasma glucose concentrations), and digestive enzymes (lipase and amylase) in plasma (*P* < 0.05). In terms of fish farm profitability, a higher net profit can be achieved by maximizing returns while minimizing costs. The histology and scanning electron microscopy (SEM) analysis of the *O. niloticus* intestines revealed significant improvements in the length and width of intestinal villi (*P* < 0.05).

**Discussion and conclusion:**

The inclusion of L-threonine (0.48g/kg) and encapsulated butyric acid (ButiPEARL) (0.3g/kg) in fish diets can serve as safe, natural, and cost-effective feed additives. It is recommended to use a combined inclusion level of these additives to enhance growth, boost immunity, support liver function, and promote intestinal development. Future research to evaluate the advantages of a dietary combination of encapsulated butyric acid and L-threonine at varying doses in aquaculture is encouraged by the findings of this investigation. However, additional research is required to ascertain these compounds' potential impact on other fish species.

## Introduction

The aquaculture sector is expanding quickly and plays a vital role in meeting the world's protein demands, especially in places like Asia and Africa where one billion people primarily rely on fish for their animal protein. [1]. The Nile tilapia, (*Oreochromis niloticus*) is an essential fish species for aquaculture. Egypt is the world's third-largest producer of tilapia, behind only China and Indonesia, and it currently accounts for around 75.54 percent of worldwide aquaculture production. [2].

Antibiotic resistance, immunological suppression, and the presence of residues of antibiotics in animal-derived products have all been linked to aquaculture's use of antibiotics [3]. Because they improve growth, immunostimulatory, and antioxidant qualities, certain immunostimulants, including butyric acid and threonine, have been added to fish feed in order to solve these issues and promote fish health and productivity. As a necessary component of body protein, threonine has been shown to enhance development when added to the diet of aquatic animals, according to the National Research Council [4]. In addition to providing an energy source and vital amino acids, lipids, vitamins, and minerals, a well-designed fish diet should increase survival and development [5]. A growing variety of fish feed additives are available that may help reduce or moderate the detrimental effects of fish diet formulations that are too high, according to Knudsen et al. [6]. These effects include immune suppression, enteritis, and decreased growth. The building blocks of proteins, amino acids, are essential for intermediate metabolism [7]. Their availability is essential in aquaculture because they have two functions: they are substrates for the synthesis of proteins and metabolic fuel for energy [8]. Among the 10 necessary amino acids found in materials for practical fish feed, threonine is often considered the most restricted amino acid, followed by lysine and methionine [9]. Protein synthesis and the production of essential metabolites including glycine, acetyl-CoA, and pyruvate are both aided by threonine. It also influences the production of gastrointestinal mucin and immune responses [10]. A feasible feed addition that has garnered a lot of attention due to its positive effects on the gastrointestinal health of humans and animals is butyrate, a short-chain fatty acid (SCFA) salt produced by bacteria fermenting undigested carbohydrates ([11]). Butyric acid in fish feed has improved carp development and feed utilization in aquaculture [12–14]. Furthermore, earlier studies by [15] have shown that when gilthead sea bream are fed butyrate, it can act as an energy source for the intestinal epithelium and cause slight increases in growth rates. Human research suggests that butyrate may affect several biological functions, such as reducing oxidative stress, enhancing the colonic defense barrier, and preventing inflammation and the onset of cancer [16, 17]. A higher dietary threonine requirement is necessary for the maintenance of mucin synthesis, a mucus glycoprotein that is found in high concentration in intestinal and skin secretions [18, 19]. As well, threonine is crucial for fish growth and fillet production since it is a protein-related precursor of non-essential amino acids like glycine and serine [10]. The dietary threonine needs for a range of fish species, such as Ictalurus punctatus and channel catfish, have been reported to be between 5 and 18 g/kg of food [20]. To the best of our knowledge, no research has examined the effects of a dietary combination of L-threonine and encapsulated butyric acid (ButiPEARL®) on the growth performance, antioxidant status, and blood biomarkers of Nile tilapia fingerlings. Therefore, the main objective of this study was to evaluate the effects of commercially encapsulated butyric acid and/or L-threonine on growth performance, antioxidant status, immune response, small intestine histomorphometry, and economic parameters of *O. niloticus* fingerlings. Therefore, the main objective of this study was to evaluate the effects of commercially encapsulated butyric acid and/or L-threonine on growth performance, antioxidant status, immune response, small intestine histomorphometry, and economic parameters of *O. niloticus* fingerlings.

## Materials and methods

### Fish maintenance

Four hundred eighty-six fingerlings of *Oreochromis niloticus*, weighing approximately 15 ± 0.5 (g) and measuring 7 ± 0.5 (cm), were obtained from a certified private fish farm in Kafr El Sheikh Governorate, Egypt. These fingerlings were transported to the Department of Aquatic Animal Medicine at Benha University, Egypt [21]. Upon arrival, Fish were housed in 750-L aerated fiberglass tanks for two weeks in order to allow them to become used to aquarium conditions and were given a commercial tilapia diet (30% CP). 27 °C is the water temperature; 6 mg/L of dissolved oxygen, 0.53 mg/L of ammonia, and a pH of 7 are the values. At a rate of thirty percent, the water was changed twice a week.

### Diets

The basal diet was formulated with the guidelines in accordance [22]. Encapsulated butyric acid used in this experiment consisted of 50% butyrate salt, obtained from Kemin Industries (1900 Scott Avenue I, Des Moines, Iowa, USA 50317), and L-threonine obtained from Lifelong Chemical Co., Ltd. with a purity of L-threonine of 98.5%. L-Threonine and butyrate were supplemented in the basal diet at appropriate concentrations as represented in Table 1. Encapsulated butyric acid was added to the diet according to the recommendation of Kemin Industries, but L-threonine was supplemented to fish at various levels. To produce the fish feeds, all ingredients were well mixed for 15 min, and then water and oil were added to form a wet, doughy mass. After that, the dough mixture was pelleted into 2 mm-diameter sinking pellets without the use of steam. Finally, using the techniques described by [23], the pellets were dried at ambient temperature and kept in sterile, clean plastic bags at −20 °C until needed.

**Table 1:** Composition and proximate analysis of basal and treated diets (g/kg)

ingredients	Group 1 (Control)	Group 2 (Butyric acid 0.3 gm	Group 3 (Threonine 0.24 gm)	Group 4 (Butyric acid 0.3 gm + Threonine 0.24)	Group 5 (Threonine 0.48 gm)	Group 6 (Butyric acid 0.3 gm + Threonine 0.48 gm)
Yellow corn	15.93	15.93	15.93	15.93	15.93	15.93
Soybean meal (44% protein)	13.6	13.6	13.6	13.6	13.6	13.6
Corn gluten (60% protein)	5	5	5	5	5	5
Fish meal	23.84	23.84	23.84	23.84	23.84	23.84
Rice bran	15	15	15	15	15	15
Wheat bran	15	15	15	15	15	15
Wheat flour	7	7	7	7	7	7
Soybean oil	2	2	2	2	2	2
Molasses	2	2	2	2	2	2
Choline chloride	0.05	0.05	0.05	0.05	0.05	0.05
Vitamin and mineral premix**	0.3	0.3	0.3	0.3	0.3	0.3
Vitamin C	0.025	0.025	0.025	0.025	0.025	0.025
Threonine	-ve	-ve	0.024	0.024	0.048	0.048
Coated Butyric acid	-ve	0.03	-ve	0.03	-ve	0.03
**Nutrient specification**						
Crude protein	30.3	30.3	30.3	30.3	30.3	30.3
Crude lipids	6.58	6.58	6.58	6.58	6.58	6.58
Crude fiber	4.94	4.94	4.94	4.94	4.94	4.94
Calcium	1.76	1.76	1.76	1.76	1.76	1.76
Total phosphorus	0.84	0.84	0.84	0.84	0.84	0.84
Lysine	1.76	1.76	1.76	1.76	1.76	1.76
Methionine	0.69	0.69	0.69	0.69	0.69	0.69
Threonine	1.16	1.16	1.4	1.4	1.64	1.64
Cystine + Methionine	1.15	1.15	1.15	1.15		1.15

### Experimental design

The fish used in this feeding trial were divided into six dietary treated groups, each having triplicates. Each replicate consisted of 27 fish. The fish in the control group (G1) were fed a basal diet, the second group (G2) fed on a basal diet supplemented with butyric acid 0.3 g/kg diet, the third group (G3) fed on a basal diet supplemented with L-threonine 0.24 g/kg diet, the fourth group (G4) fed on a basal diet supplemented with butyric 0.3 g/kg + L-threonine 0.24 g/kg diet, the fifth group (G5) fed on a basal diet supplemented with L-threonine 0.48 g/kg diet, and the sixth group (G6) fed on a basal diet supplemented with butyric 0.3 g/kg + L-threonine 0.48 g/kg. The feeding was done twice daily, with the food given being 5% of the fish's body weight. This feeding regimen was followed for 42 days. The water parameters were maintained at appropriate levels throughout the experiment, similar to the acclimatization period. All procedures conducted in this study were in accordance with the approved guidelines for the use of laboratory animals set by the Experimental Animal Use Committee at Benha University, Egypt.

### Evaluation of growth performance and hepatosomatic indices

Several metrics were used to evaluate the fish's growth performance. The fish were weighed and counted at the start of the experiment, three weeks into it, and at the end of the six-week period. The fish were fasted for six hours and dried with sterile filter paper to remove any remaining water before being weighed. Twice a day, between 9:00 am and 5:00 pm, the fish were hand fed at a rate of 5% of their body weight. A number of metrics were computed in order to assess growth performance. They were as follows: initial body weight, final body weight, weight gain (WG), which was measured by subtracting the average initial weight from the average final body weight, and weight gain rate, which was computed by dividing the average body weight by the average initial body weight [24]. Using the formula (Ln Final weight—Ln Initial weight) divided by the number of trial days multiplied by 100, the specific growth rate (SGR) was calculated. The fish's initial and final lengths were also measured, and the average initially length was subtracted from the average final length to determine the length increase. By dividing the amount of feed provided by the weight increase, the feed conversion ratio (FCR) was calculated using the procedure outlined by [25]. Additionally, the hepatosomatic index (HSI) was computed by dividing the liver weight (g) by the body weight (g) and then multiplying the result by 100. A conventional procedure described by [26] was used to determine this index.

### Determining measurements of the profitability

The cost and return parameters associated with each 1000 fish are calculated in Egyptian pounds (EGP) and then converted to dollars ($) (where each dollar is equivalent to 15.65 EGP) to estimate profitability measures. Total cost (TC), total variable cost (TVC), and total fixed cost (TFC) are the costs taken into account in this study. The following formulas were utilized in the computation of these expenses: The total of total variable cost (TVC) and total fixed cost (TFC) equals total cost (TC). To calculate TFC, the equipment value was divided by the equipment age. The equipment depreciation value per fish was then calculated by dividing production by the number of fish each year. TFC per was then determined by multiplying this number by 1000. TFC per 1000 fish was then computed by multiplying this amount by 1000. TVC comprises the cost of feed (calculated by multiplying the cost of one gram of each experimental diet by the feed consumption per gram), labor, water, electricity, and disinfectant, as well as fish per thousand fingerlings (obtained by multiplying the value of each fingerling by 1000). Net profit (NP) and total returns (TR) are two return criteria considered in this analysis, where TR is equal to the market price per gram times the weight of 1000 fish per gram. NP = Total return—Total expense [27].

### Sampling

Blood samples were obtained from the fish after the 3rd and 6th weeks of the feeding trial. A 1-mL heparinized syringe was used for collection, with 6 fish per group and 3 fish per tank. Before blood collection, the fish were subjected to euthanasia using 250 ppm of tricaine methanesulfonate (MS222) in water obtained from Syndel Laboratories in British Columbia. The collected blood specimens were centrifuged at 2,058 xg at 4°C for 5 min to separate the plasma. The plasma was subsequently analyzed for glucose levels and digestive enzymes (lipase and amylase). After plasma collection, liver tissues were collected in phosphate-buffered saline (PBS) with a pH of 7.4. These liver tissues assessed nitrous oxide, superoxide dismutase, catalase, and malondialdehyde (colorimetric assay kits ab65354). Blood samples without anticoagulants were also collected for serum samples, following the same procedures described by Youssef et al. [28]. These serum samples were analyzed to determine total protein levels, albumin, globulin, IgM (ELISA kits) (My BioSource Inc.), and liver enzymes (ALT, AST) (liver function test kits, Amazon).

#### Histology and electron microscope samples

For this study, three fish were selected from each tank in the contro l, 2, 3, 4, 5, and 6 groups. The fish were euthanized, and their ventral body wall was dissected. The entire gastrointestinal tract of the twelve fish was removed and thoroughly rinsed with distilled water. Specific sections of the intestine were then extracted from the middle portion for further analysis.

### Immunological parameters

The technique of [29] was used to determine the serum levels of total protein albumin. By deducting the albumin value from the total blood protein, the globulin levels were determined. Using ELISA kits, the serum IgM level was determined spectrophotometrically (Cusabio Biotech Co. Ltd, USA). At 570 nm, nitrous oxide was detected using spectrophotometry. Nitrite oxidation, which is directly proportional to NO concentration, was used to measure the levels of nitrous oxide. According to the approach stated by [30], this was accomplished by mixing an 85-μl sample with an equivalent volume of Griess reagent (Sigma-Aldrich, USA).

### Antioxidants and biochemical measurements

The levels of catalase (CAT) and superoxide dismutase (SOD) were assessed to determine their enzymatic activity using established methods described by [31]. SOD activity was determined by measuring the enzyme's ability to inhibit phenazine methosulphate using nitro blue tetrazolium dye, an indicator with a wavelength of 340 nm. By tracking the drop in hydrogen peroxide concentration at 540 nm, CAT activity was determined. MDA serves as a useful oxidative stress biomarker. The MDA concentration is calculated using the technique of [32] (nmol/mg protein). The absorbance of the conjugate produced by the reaction of MDA with thiobarbituric acid (TBA) at 532 nm was measured in comparison to a reagent blank. Spectrophotometric measurements of glucose concentration, aspartate aminotransferase (AST), and alanine aminotransferase (ALT) were performed 340 nm. The decrease in absorbance was monitored kinetically over 5 min, following the methods described by [13, 33].

### Digestive enzymes

Using diagnostic reagent kits and the defined technique supplied by Cusabio Biotech Co. Ltd., China, the enzyme activity of lipase and amylase in plasma were evaluated. One international unit (UI) of amylase is defined as the quantity of enzyme required to hydrolyze one micromole of substrate per minute under normal circumstances. The activity was expressed as mU per protein milligram. As demonstrated by [34], a kinetic test was used to determine lipase, measuring the rise in absorbance at 580 nm.

In summary, samples that had been homogenized and kept at −80 °C were thawed on ice and combined with a buffer that contained the following values (all in milligrams): 0.05 CaCl2, 30 mannitol, 1 mg·L − 1 colipase (pH 8.3), 3.6 taurodeoxycholate, 0.9 deoxycholate, 0.8 tartrate, 0.12 DGGR (1,2-Di-O-lauryl-rac-glycero-3-(glutaric acid 6-methylresorufin ester) as substrate), 0.05 CaCl2, and 30 mannitol. The linear zone was identified between 10 and 20 min of reaction time, and absorbance was recorded every minute for 45 min. The standard, lipase (Sigma Aldrich, Spain, L0382, 33,944 U·mg protein − 1, 22,980 U·mg solid − 1), was first diluted to 20 U·mL − 1. Under normal circumstances, one international unit (IU) is the quantity of enzyme required to hydrolyze one micromole of substrate per minute. mU per milligram of protein was used to determine lipase activity.

### Light microscopy (LM)

After the intestine was removed right away, pieces of it were carefully preserved in a 10% buffered neutral formalin solution. The pieces then went through a number of preparation processes, such as being dehydrated in alcohol, cleared in xylene, embedded in paraffin, and lastly sliced into thin, 5 μm-thick slices. Hematoxylin and eosin (H&E) was used to stain the sections. Methods and procedures for staining and fixing were done in accordance with [35]. A Leica DM 3000 LED computerized light microscope was used to view the stained sections.

### Scanning electron microscopy (SEM)

In order to removal of food particles, the gut mucosal surface was gently cleaned using a standard saline solution. Fresh specimens were then cut into thin slices and kept for three hours at 4 degrees Celsius in a glutaraldehyde solution with a pH of 7.4. The samples were then washed three times in phosphate-buffered saline (PBS) for 10 min each. Following a series of increasing ethyl alcohol percentages (30%, 50%, 70%, 90%, and 100% alcohol) for dehydration, they were fixed in 1% osmium tetraoxide for half an hour at room temperature. After injecting acetone into the tissues, each solution was applied for half an hour. A Samdri-PVT-3B® critical point drying equipment (Tou-simis, Rockville, USA) was used to dry the samples using liquid CO2. This was followed by mounting them on aluminum stubs and applying a 0.04 lm layer of gold using a sputter-coating equipment (JFC-1100 E). Lastly, a Jeol-JSM-5300 LV scanning electron microscope (Tokyo, Japan) operating at 20 kV was used to view the coded specimens in the electron microscopy unit at Alexandria University in Egypt's Faculty of Science.

### Statistical analysis

Tukey's tests, one-way and two-way analysis of variance (ANOVA), and the computer application SPSS/PC + "version 23 [36]" were used to examine the statistical significance between the control and treatment groups. The data was presented using the mean ± SEM. Notable *p*-values were those that were less than 0.05 (*P* < 0.05).

## Results

### Growth performance and profitability measures

The growth performance parameters presented in Table 2 differed significantly (*P* = 0.00) among different groups. In its diet, the fish group supplemented with encapsulated butyric acid (ButiPEARL) 0.3 g/kg feed mixed with L-threonine 0.48 g/kg feed recorded the highest values throughout the experiment. Regarding the first three weeks of rearing, the fish group supplemented with encapsulated butyric acid (ButiPEARL) 0.3 g/kg feed mixed with L-threonine 0.48 g/kg feed in its diet showed the best length gain, weight gain, WGR, SGR, and FCR, while they were the worst for the control group. On the other hand, during the last three weeks of rearing, the weight gain was the highest for the L-threonine 0.48 g/kg feed-supplemented group, and it was the lowest for the ButiPEARL 0.3 g/kg feed. Regarding the WGR, SGR, and FCR during the last three weeks, they were the highest for the L-threonine 0.24 g/kg feed supplemented group, while the ButiPEARL 0.3 g/kg feed mixed with the L-threonine 0.48 g/kg feed supplemented group showed the lowest WGR and SGR, and the ButiPEARL 0.3 g/kg feed supplemented group showed the worst FCR.

**Table 2:** Effect of different dietary levels of L-threonineon and encapsulated butyric acid (ButiPEARL) on serum biomarkers and antioxidant status of Nile tilapia fingerlings at three and six weeks of experiment

	G1		G2		G3		G4		G 5		G 6		MSE	P values		
Weeks	**3rd**	**6th**	**3rd**	**6th**	**3rd**	**6th**	**3rd**	**6th**	**3rd**	**6th**	**3rd**	**6th**		**T**	**P**	T * P
SOD (nM/g)	8.12^e^	13.54^d^	12.45^d^	14.99^ cd^	12.99^d^	16.16^c^	14.11^d^	17.55^c^	18.77^c^	33.01^b^	32.12^b^	46.17^a^	0.190	0.000	0.000	0.000
NO (µM/g)	12.04^e^	15.67^d^	13.43^e^	18.40^d^	13.92^e^	19.33^d^	21.22^d^	30.11^c^	28.55^c^	43.06^b^	44.28^b^	63.41^a^	0.355	0.000	0.000	0.000
CAT (nM/g)	10.37^d^	15.36^c^	11.49^ cd^	20.30^c^	12.15^d^	20.30^c^	13.48^d^	22.21^c^	24.84^c^	32.75^b^	24.85^c^	43.99^a^	0.230	0.000	0.000	0.000
MDA (nM/g)	21.15^a^	18.86^b^	19.65^abc^	17.65^c^	18.50^b^	15.77^d^	17.98^c^	14.03^e^	13.08^c^	9.86^f^	11.61^ cd^	5.06^ g^	0.123	0.000	0.000	0.001
T. protein (mmol/L)	3.13	3.06^d^	3.71	3.75^ cd^	4.07	4.28^bcd^	3.91	5.07^bc^	4.50	5.85^b^	4.86	7.49^a^	0.130	0.000	0.002	0.045
Albumin (mmol/L)	1.77^b^	2.07^b^	1.90^ab^	2.45^b^	2.11^ab^	2.52^ab^	2.16^ab^	2.71^ab^	2.49^a^	3.01^ab^	2.31^ab^	4.20^a^	0.113	0.035	0.005	0.377
Globulin (mmol/L)	1.37	0.99	1.81	1.30	1.96	1.76	1.75	2.93	2.01	2.83	2.54	3.29	0.165	0.060	0.406	0.559
IgM (µmol/L)	7.37^d^	7.94^d^	7.25^d^	8.94^d^	8.22^ cd^	11.05^c^	9.28^bc^	12.05^bc^	10.58^b^	13.35^ab^	12.28^a^	15.17^a^	0.131	0.000	0.000	0.097
AST (U/L)	27.83^a^	126.00^a^	26.33^a^	94.00^ab^	22.33^ab^	87.33^ab^	17.07^ab^	69.00^ab^	14.43^b^	48.67^b^	13.87^b^	37.60^b^	3.320	0.002	0.000	0.047
ALT (U/L)	14.53^a^	40.94^a^	14.37^a^	41.59^a^	13.07^a^	37.79^ab^	12.77^a^	27.37^ab^	11.13^ab^	19.48^b^	9.44^b^	21.07^b^	1.213	0.009	0.000	0.125
Glucose (mg/dl)	73.00^c^	87.00^d^	82.33^c^	102.00^d^	91.00^bc^	135.00^c^	106.67^bc^	158.67^bc^	138.33^b^	180.33^b^	206.00^a^	221.00^a^	2.797	0.000	0.000	0.228
Lipase (U/L)	1.27^c^	1.98^c^	1.71^bc^	2.25^c^	1.91^bc^	2.71^c^	2.12^bc^	3.58^b^	2.56^b^	5.00^a^	4.18^a^	5.83^a^	0.063	0.000	0.000	0.001
Amylase (U/L)	30.82^c^	45.94^d^	35.79^bc^	60.84^c^	39.57^bc^	76.24^b^	46.64^bc^	77.25^b^	49.46^b^	80.05^b^	78.40^a^	89.77^a^	0.773	0.000	0.000	0.000

Values are expressed as mean value (n = 3) ± SE. Mean values with different superscript letters are significantly different (P < 0.05). SOD, superoxide dismutase; CAT,catalase; NO, nitric oxide; MDA, malondialdehyd; IgM, Immunoglobulin M;AST, Aspartate aminotransferase; ALT, alanine aminotransferase

G1: Control group, G2: Butyric acid 0.3 gm supplemented group, G3: Threonine 0.24 gm supplemented group,G4: Butyric acid 0.3 gm + Threonine 0.24 gm supplemented group, G5: Threonine 0.48 gm supplemented group, G6: Butyric acid 0.3 gm + Threonine 0.48 gm supplemented group

At the end of the experiment, all treated groups showed a better result than the control group. The group supplemented with ButiPEARL 0.3 g/kg feed mixed with L-threonine 0.48 g/kg feed significantly improved the growth rates and feed utilization efficiency of Nile tilapia; it was the most efficient group as it had the best final body weight, length, length gain, weight gain, WGR, SGR, and FCR. In responding to liver weight and HSI, they were the highest for the L-threonine 0.24 g/kg feed-supplemented group, while they were the lowest for the control. Regarding the profitability of fish farms, the higher the return with minimum cost, the higher the net profit. In our study, the total cost was nearly similar in all groups, as shown in Table 3. In contrast, the total return significantly differed (*P* = 0.00) among different groups. It was the highest for the group that supplemented with ButiPEARL 0.3 g/kg feed mixed with L-threonine 0.48 g/kg feed in its diet, followed by the L-threonine 0.48 g/kg feed supplemented group ($108.84). It was the lowest for the control group. Consequently, the net profit was the highest for the group that supplemented with ButiPEARL 0.3 g/kg feed mixed with L-threonine 0.48 g/kg feed in its diet, followed by the L-threonine 0.48 g/kg feed supplemented group, while it was the lowest for the control group.

**Table 3:** Effect of different dietary levels of L-threonineon and encapsulated butyric acid (ButiPEARL) on growth performance,HSI, and feed utilization of Nile tilapia (*Oreochromis niloticus*) fingerlings at three and six weeks of experiment

	G1		G2		G 3		G 4		G5		G6		MSE	*P* values		
Weeks	**3rd**	**6th**	**3rd**	**6th**	**3rd**	**6th**	**3rd**	**6th**	**3rd**	**6th**	**3rd**	**6th**		**T**	**P**	T * P
Length (cm)	10.6^d^	12.8^c^	12.36^c^	13.3^c^	12.38^c^	14.42^b^	13.82^b^	15^a^	14.24^b^	15.5^a^	14.52^b^	15.55^a^	0.087	0.000	0.000	0.209
Length gain (%)	3.7^c^	2.2^d^	5.46^b^	0.94^e^	5.22^b^	2.03^d^	6.98^a^	1.18^de^	7.04^a^	1.26^de^	7.68^a^	1.03^e^	0.140	0.014	0.000	0.000
Weight (g)	21.96^f^	35.0^d^	28.26^e^	39.28^d^	28.33^e^	45.72^c^	35.48^d^	51.2^bc^	38.94^cd^	56.78^ab^	42.28^c^	58.03^a^	0.691	0.000	0.000	0.151
WG (g)	7.36^d^	13.04^c^	13.56^c^	11.02^ cd^	13.33^c^	17.39^bc^	20.65^bc^	15.72^c^	24.14^ab^	17.84^bc^	27.53^a^	15.75^c^	0.849	0.002	0.164	0.051
FCR (%/day)	2.736^a^	2.02^ab^	1.438^bc^	2.83^a^	1.53^b^	1.56^b^	0.93^cd^	1.78^ab^	0.82^cd^	1.64^b^	0.71^d^	1.86^ab^	0.110	0.001	0.003	0.004
WGR (%)	50.782^ cd^	59.79^c^	92.07^c^	40.77^d^	89.35^c^	62.51 ^cd^	139.35^b^	45.15^d^	163.68^ab^	48.16^d^	187.03^a^	39.27	3.825	0.002	0.000	0.000
SGR (%/day)	1.938^c^	2.22^bc^	3.1^b^	1.57^d^	3.01^b^	2.28^bc^	4.15^a^	1.74^cd^	4.57^a^	1.83^c^	4.99^a^	1.54	0.106	0.009	0.000	0.000
Liver weight (g)	0.38^d^	0.62^bc^	0.58^cd^	1.0^b^	0.57 ^cd^	2.45^a^	0.83^bc^	1.37^ab^	0.87^bc^	1.46^ab^	0.99^b^	2.06^a^	0.059	0.004	0.000	0.373
HSI (%)	1.742^d^	1.78^cd^	2.026^c^	2.52^bc^	2.02^c^	5.22^a^	2.33^c^	2.68^bc^	2.21^c^	2.57^bc^	2.33^c^	3.53^b^	0.114	0.271	0.046	0.470

Values are presented with different superscripts within each row indicate significant differences (*p* < .05)

G1: Control group, G2: Butyric acid 0.3 gm supplemented group, G3: Threonine 0.24 gm supplemented group,G4: Butyric acid 0.3 gm + Threonine 0.24 gm supplemented group, G5: Threonine 0.48 gm supplemented group, G6: Butyric acid 0.3 gm + Threonine 0.48 gm supplemented group, WG: weight gain, FCR: Feed conversion ratio, HIS: Hepatosomatic index, FCR: Feed conversion ratio, WGR, Weight gain ratio, SGR: Specific growth rate

### Immunological parameters

In the current investigation, the efficacy of dietary L-threonine and encapsulated butyrate was assessed after three weeks and six weeks of feeding trials (Table 4). Total protein demonstrated a non-significant elevation in the treated groups compared to the control in the third week of the experiment. But at the end of the experiment, it showed a significant increase (P < 0.05), especially in the supplemented groups 4, 5, and 6. Nonetheless, albumin levels were significantly higher in treated groups at half and end of the experiment, especially groups 5 and 6 compared to the control group. Regarding the globulin value, no statistically significant alteration was observed in the dietary treated groups compared to the control group.

**Table 4:** Effect of different dietary levels of L-threonine and encapsulated butyric acid (ButiPEARL) on growth performance and profitability measures of *O. niloticus* at the end of the experiment

	G1	G2	G3	G4	G5	G6	MSE	*P* value
Initial wt (g)	14.6	14.7	15.00	14.83	14.80	14.75	0.243	0.906
Initial length (cm)	6.9	6.9	7.17	6.83	7.20	6.83	0.200	0.649
Total length gain (%)	5.9^d^	6.4^cd^	7.25^bc^	8.17^ab^	8.30^ab^	8.72^a^	0.300	0.000
Total feed intake (g)	44.2	44.2	44.20	44.20	44.20	44.20	0.000	1.000
Total WG (g)	20.40^e^	24.58^de^	30.72^cd^	36.37^bc^	41.98^ab^	43.28^a^	1.697	0.000
Total FCR (%/day)	2.214^a^	1.828^b^	1.47^c^	1.24^cd^	1.06^d^	1.02^d^	0.087	0.000
Total WGR (%)	140.38^d^	167.714^cd^	205.36^bc^	246.38^ab^	284.09^a^	294.58^a^	13.998	0.000
Total SGR (%/day)	2.078^d^	2.336^cd^	2.65^bc^	2.94^ab^	3.20^a^	3.26^a^	0.105	0.000
Kg feedcost (EGP)	10	10.05	10.01	10.06	10.02	10.07	0.000	
G feedcost ((EGP)	0.01	0.01	0.01	0.01	0.01	0.01	0.000	0.000
Management cost/1000 (EGP)	100	100	100.00	100.00	100.00	100.00	0.000	
TFC/1000 (EGP)	30	30	30.00	30.00	30.00	30.00	0.000	
Feed cost /1000 (EGP)	442^f^	443.99^c^	442.53^e^	444.52^b^	443.06^d^	445.05^a^	0.000	0.000
TVC/1000 (EGP)	542	543.99	542.53	544.52	543.06	545.05	0.000	
TC/1000 (EG	572	573.99	572.53	574.52	573.06	575.05	0.000	
TR/1000 (EGP)	1050.06^d^	1178.4^d^	1371.55^c^	1536.00^bc^	1703.40^ab^	1740.85^a^	47.094	0.000
NP/1000 (EGP)	478.06^d^	604.41^d^	799.02^c^	961.48^bc^	1130.34^ab^	1165.80^a^	47.094	0.000
TVC/1000 ($)	34.63	34.76	34.67	34.79	34.70	34.83	0.000	
TFC/1000 ($)	1.92	1.92	1.92	1.92	1.92	1.92	0.000	
TC/1000 ($)	36.55	36.68	36.58	36.71	36.62	36.74	0.000	
TR/1000 ($)	67.098^d^	75.298^d^	87.64^c^	98.15^bc^	108.84^ab^	111.24^a^	3.009	0.000
NP/1000 ($)	30.548^d^	38.622^d^	51.06^c^	61.44^bc^	72.22^ab^	74.49^a^	3.009	0.000
Feed cost /1000 ($)	28.24^f^	28.37^c^	28.28^e^	28.40^b^	28.31^d^	28.44^a^	0.000	0.000

G1: Control group, G2: Butyric acid 0.3 gm supplemented group, G3: Threonine 0.24 gm supplemented group,G4: Butyric acid 0.3 gm + Threonine 0.24 gm supplemented group, G5: Threonine 0.48 gm supplemented group, G6: Butyric acid 0.3 gm + Threonine 0.48 gm supplemented group, WG: Weight gain, FCR: Feed conversion ratio, WGR: Weight gain ratio, SGR: Specific growth rate, TVC: Total variable cost, TFC: Total fixed cost, TC: Total cost, TR: Total return, NP: Net profit

IgM showed a significant increase (*P* < 0.05) in the supplemented groups relative to the control group. The higher values of IgM were observed in the group fed on a diet supplemented with 0.48 g of threonine and 0.3 g of sodium butyrate at the third week and six weeks. Also, nitric oxide demonstrated a significant elevation (*P* < 0.05) in the supplemented groups compared to the control group, and the highest level of NO (*P* < 0.05) was reported in G6, which received a diet containing (0.48 g of threonine with 0.3 g sodium butyrate).

Also, nitric oxide demonstrated a significant elevation (*P* < 0.05) in the supplemented groups compared to the control group, and the highest level of NO (*P* < 0.05) was reported in G6, which received a diet containing (0.48 g of threonine with 0.3 g sodium butyrate).

### Antioxidants and biochemical measurements

The serum levels of ALT and AST, as presented in Table 4, exhibited a non-significant decline compared to the control group at both the midpoint and end of the experiment. Conversely, plasma glucose concentrations displayed a significant elevation (*P* < 0.05) in the dietary supplemented groups; the lowest level was observed in the control group. Notably, group 6 exhibited the highest results in this regard. Superoxide dismutase (SOD) and catalase enzymes were assessed, and the results are presented in Table 4. The findings indicate a significant increase (*P* < 0.05) in all supplemented groups, particularly in G6, which was fed on a diet supplemented with butyric acid (0.3 g) and threonine (0.48 g). Notably, a significant change was observed at the end of the experiment compared to the halfway point. Furthermore, the malondialdehyde (MDA) levels exhibited a notable decrease (*P* < 0.05) in both sampling periods compared to the control group (Table 4).

### Digestive enzymes

Plasma amylase and lipase showed a notable elevation (*P* < 0.05) at the half and end of the experiment when compared to the control group (Table 4); also, there is a significant difference between their results at the end of the third week and sixth weeks. Groups 5 and (6) showed the highest levels, respectively, in comparison with the control group.

### Histology and scanning electron microscopy (SEM)

**Fig. 1:**
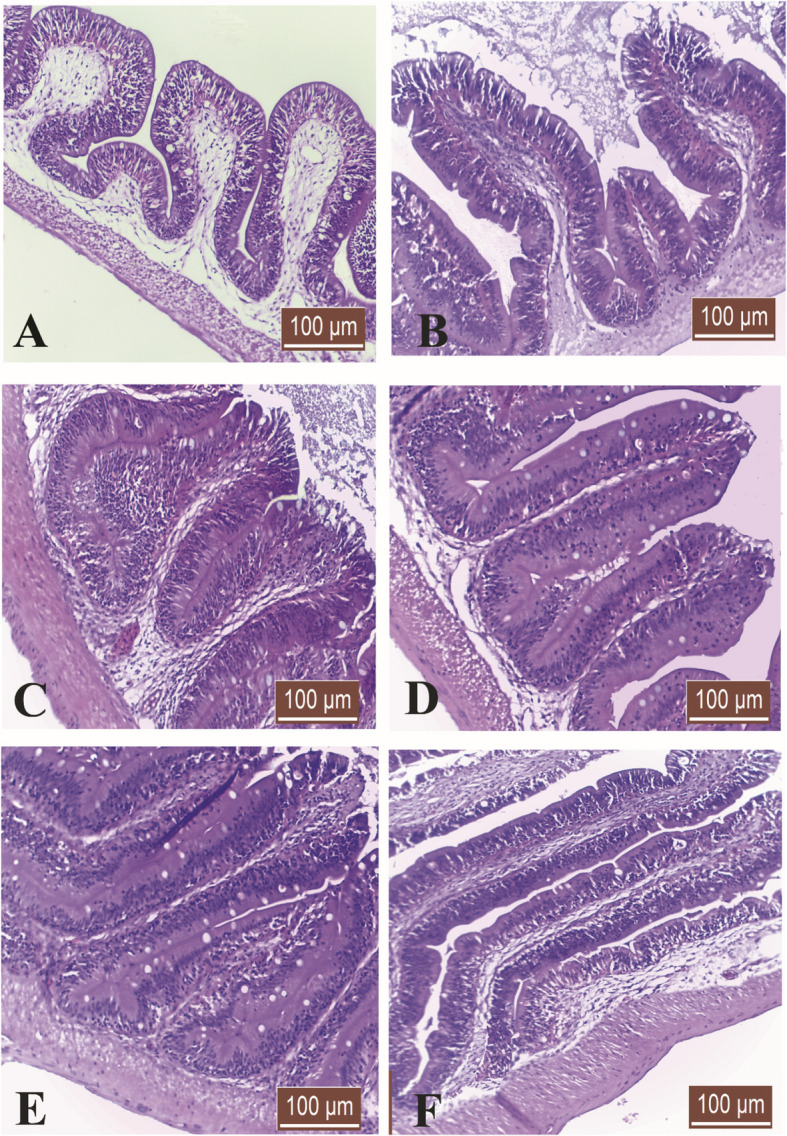
Histological sections of Nile tilapia intestinal villi in group 1 (**A**) (control) and treated groups G2 (**B**), G3 (**C**), G4 (**D**), G5 (**E**) and G6 (**F**) showing gradual increase in the length of intestinal villi during study with noticeable branching of intestinal villi in last stage. H&E stain, bar indicates magnification

**Fig. 2:**
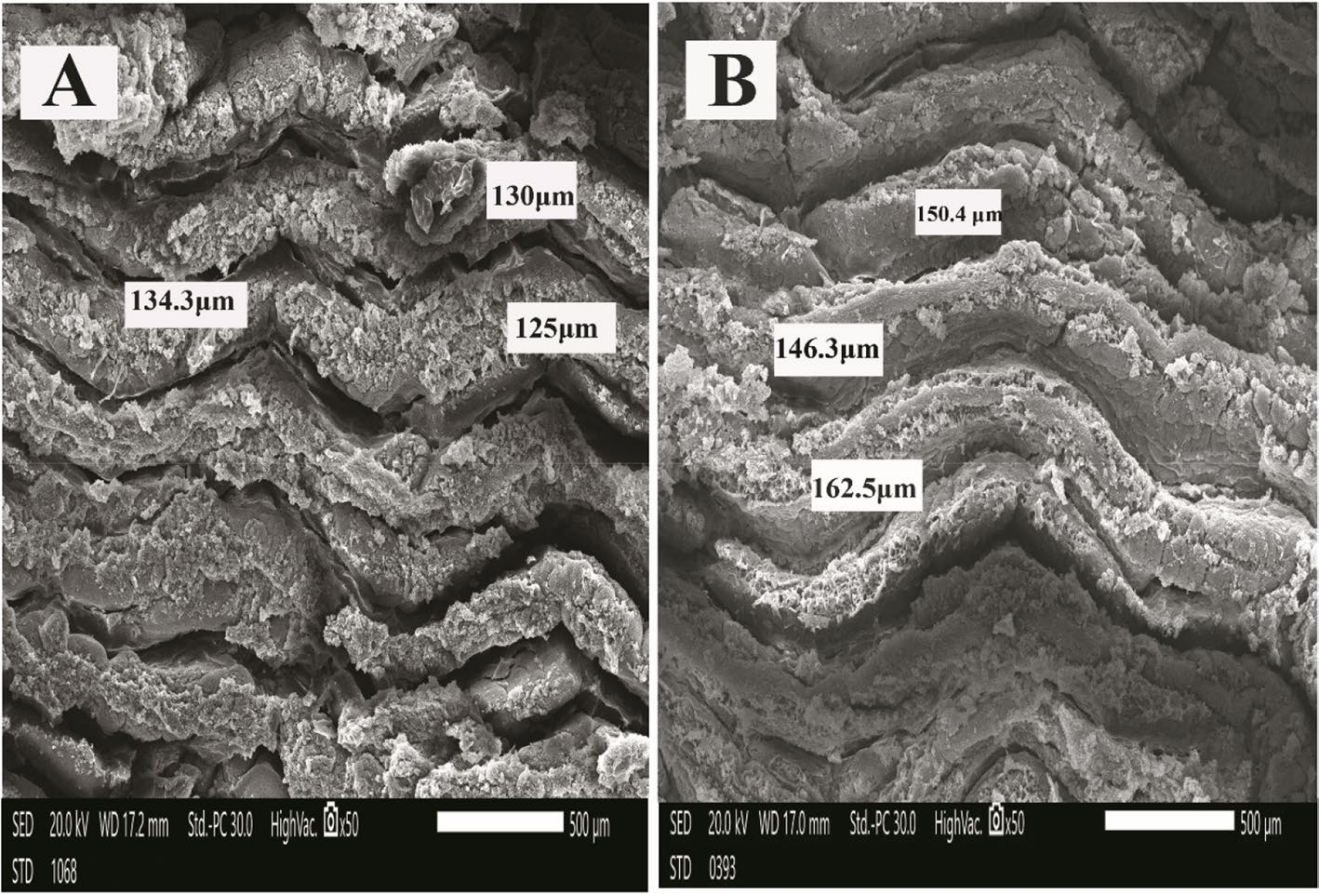
Scanning electron micrograph of Nile tilapia intestinal villi of G1 (control) (**A**) and G2 (**B**) that fed with (Butyric acid 0.3 g), bar indicates magnification

**Fig. 3:**
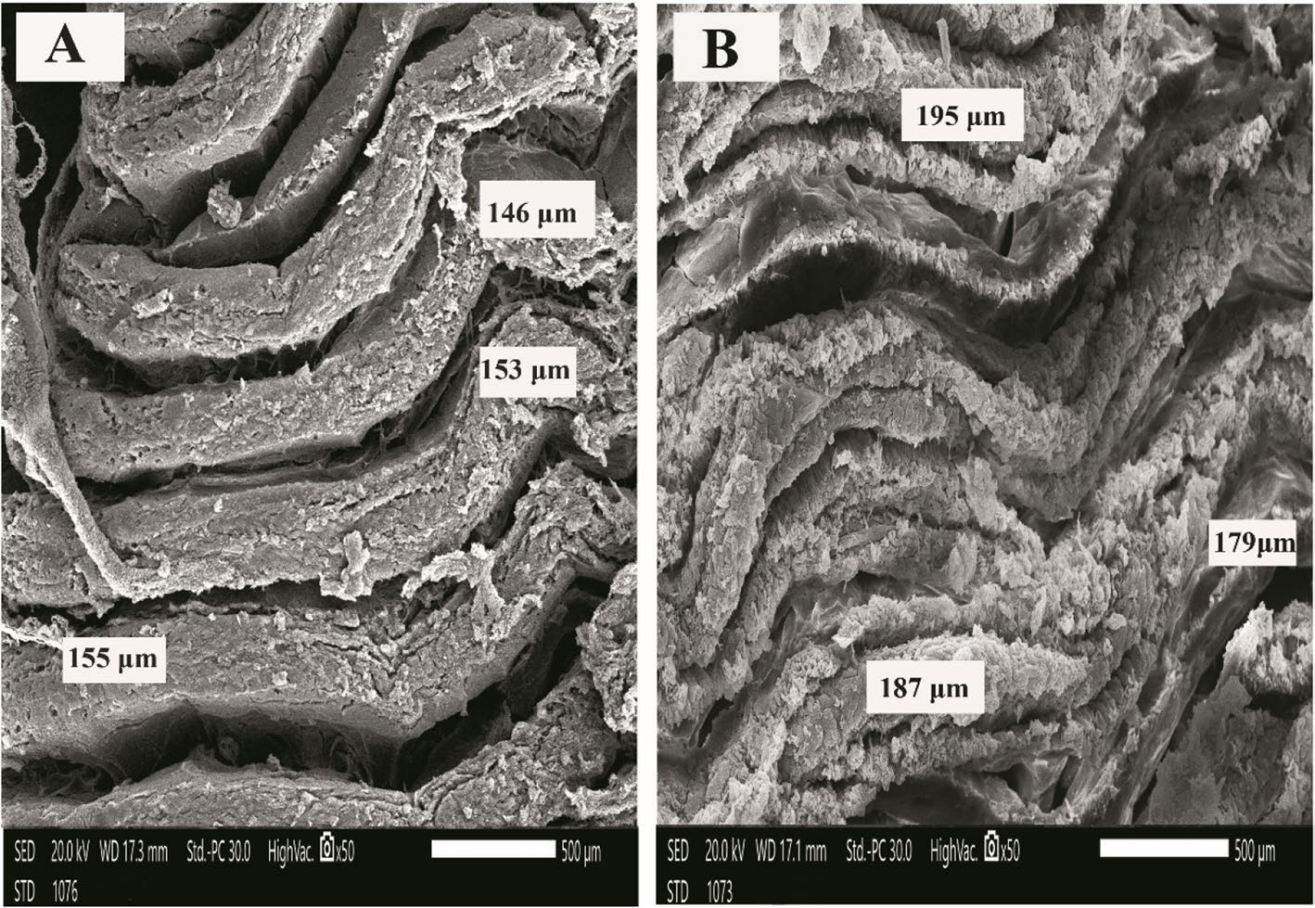
Scanning electron micrograph of Nile tilapia intestine of G3 (**A**) that fed with (threonine 0.24g) and G4 (**B**) that fed with (butyric acid 0.3 g + threonine 0.24g), bar indicates magnification

**Fig. 4:**
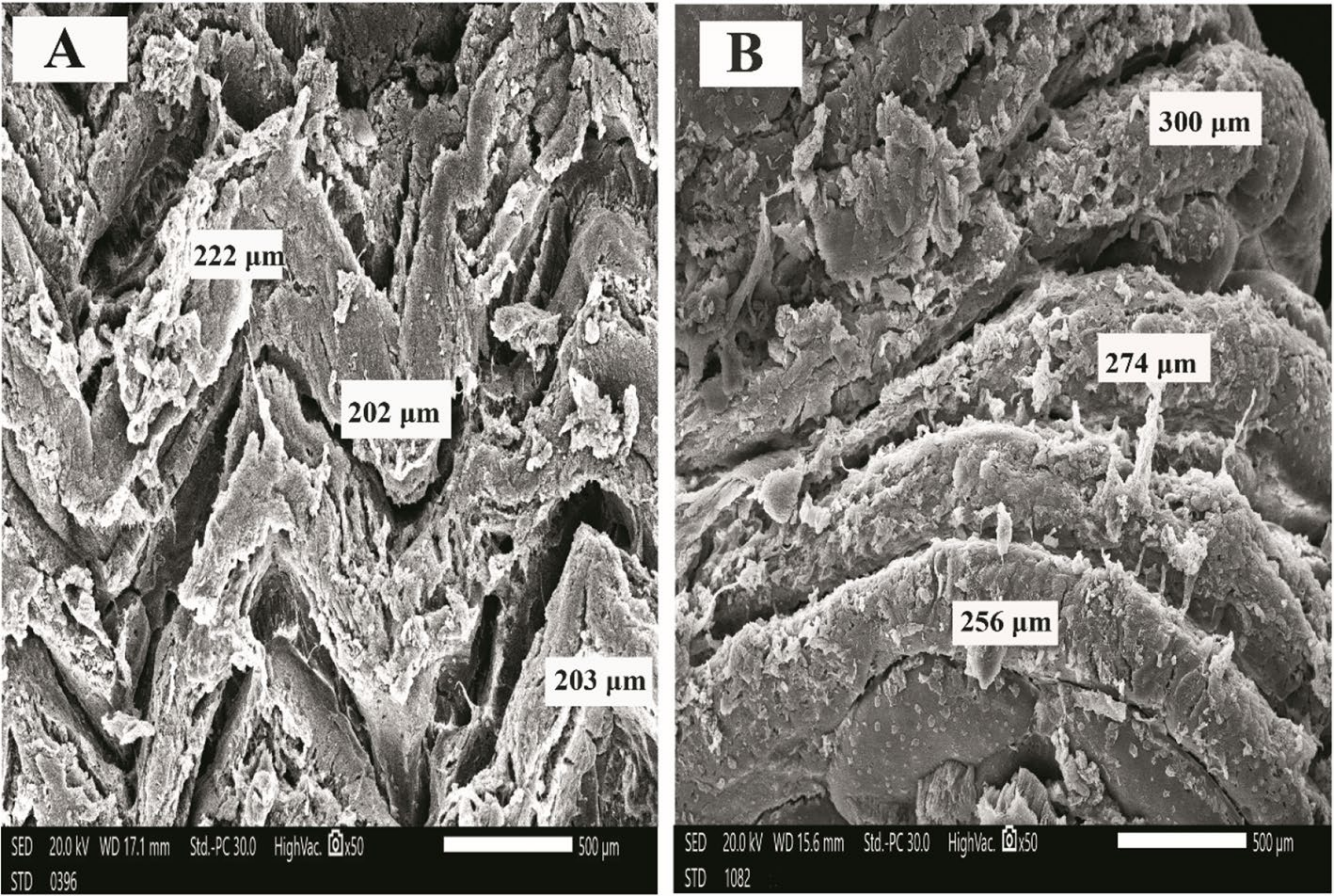
Scanning electron micrograph of Nile tilapia intestine of G5 (**A**) that fed with (threonine 0.48g) and G6 (**B**) that fed with (butyric 0.3g & threonine 0.48g), bar indicates magnification

As shown in Fig. 1, Intestinal villi showed a gradual increase in the length of intestinal villi of *O. niloticus* during the study, with noticeable branching of intestinal villi in the group (6). The scanning electron microscopy (SEM) of *O. niloticus* intestine is shown in Fig. 2, 3 and 4. The intestinal villi from fish (control group) were the smallest of all, and the walls were the thinnest as compared to those from groups 2, 3, 4, 5, and 6 (Table 5). In summary, the intestinal villi of *O. niloticus* exhibited progressive growth in both size and thickness in response to the inclusion of dietary supplements, as depicted in Fig. 2, 3 and 4. The intestinal villi from group 1 (the control group) were the shortest of all (*P* < 0.05) as compared to those from groups 2, 3, 4, 5, and 6 (Table 5). Gradual increase of the length (*P* < 0.05) of the intestinal villi of *O. niloticus* in dietary treated groups.

**Table 5:** Results of scanning electron micrograph of tilapia intestinal villi (width & length) among different feeding groups at the end of the experiment

	G1	G2	G3	G4	G5	G6	MSE	*P* value
villi width (µm)	214.25^d^	309.23^c^	347.91^c^	435.71^b^	437.91^b^	758.33^a^	12.35	0.000
villi Length (µm)	129.15^c^	153.11^c^	151.45^c^	187.76^b^	209.34^b^	277.40^a^	5.90	0.000

Values are expressed as mean value (*n* = 3) ± SE. Mean values with different superscript letters are significantly different (*P* < 0.05)

G1: Control group, G2: Butyric acid 0.3 gm supplemented group, G3: Threonine 0.24 gm supplemented group,G4: Butyric acid 0.3 gm + Threonine 0.24 gm supplemented group, G5: Threonine 0.48 gm supplemented group, G6: Butyric acid 0.3 gm + Threonine 0.48 gm supplemented group

## Discussion

Concerning the growth performance parameters, published data on the effect of encapsulated butyric acid (ButiPEARL) 0.3 gm/kg feed mixed with L-threonine 0.48 gm/kg feed on the growth performance and intestinal metabolism of Nile Tilapia fish are limited; however, in our study, this mix recorded the best result. As it had the best final body weight, length, length gain, weight gain, WGR, SGR, and FCR, at the same time the groups that supplemented with ButiPEARL alone or L-threonine alone showed better growth performance parameters compared with the control one. Our result aligns with [15], who concluded that a butyrate-supplemented diet increased the weight of juvenile sea bream compared to the control one, which might be because the butyrate enhanced the availability of nucleotide derivatives and essential amino acids. At the same time, the energy provision for enteric cells is improved by a reduction in amino acid and glucose oxidation associated with using butyrate as fuel. Additionally, butyrate increased the activity of transmethylation. Also, the dietary inclusion of a protected form of butyric acid in aquafeeds improved growth performance and fish productivity [14]. That might be due to increased butyrate efficiency when fed in an encapsulated form (ButiPEAR) [37].

Regarding the supplementation of threonine alone, our result aligns with [38]**,** who concluded that dietary supplementation of threonine increased the growth rate of grass carp. Additionally, the supplementation of L-threonine (13.5 g/kg feed) for fish improved the growth performance parameters significantly (p < 0.05) in comparison with the control one [39]. Our result disagreed with [40], who recorded a non-significant difference in dietary threonine supplementation.

Concerning the economic analysis, although there was a relatively higher feed cost and total costs for treated groups compared with the control one, they showed a significant increase in the total return and net profit than the control due to better FCR with higher weight gain. Our economic analysis showed the highest profitability was for the fish group that supplemented with ButiPEARL 0.3 g/kg feed mixed with L-Threonine 0.48 g/kg feed in its diet. Our result agreed with [41], who concluded that cost efficiency reduces with the feed conversion ratio. Our study suggests that there are still opportunities to enhance feed efficiency and profitability in Egyptian fish farms.

In the present investigation, a fish-fed diet containing 0.48 g/kg L-threonine and 0.3 g/kg, especially in the mixed group [7], significantly enhanced biochemical, immunological, and antioxidative parameters and digestive enzymes. The activity of antioxidative enzymes, such as superoxide dismutase, catalase, and glutathione peroxidase, increased, indicating a reduction in reactive oxygen species and harmful free radicals [42].

The supplementation of L-threonine and sodium butyrate significantly increased the activity of CAT compared with the control, which is a marker of lipid oxidation [43]. Dietary threonine levels that are appropriate may raise CAT activity and lower MDA [44]. The SOD enzyme transforms the superoxide anion to hydrogen peroxide, which is further used as a substrate by GPx and CAT enzymes [45]. This finding is consistent with previous studies by [46] for hybrid catfish and [47]for blunt snout bream. A recent study on rainbow trout [48], found that butyric acid strengthens the activity of antioxidant enzymes like CAT and SOD. In the present study, the least MDA was observed in the higher dose of L-threonine (0.48 g) and 0.3 g sodium butyrate within the diet, which might be because of a vital role of MDA as a margin for collapsed lipid molecules inside the fish tissues [49]. The same results for MDA in juvenile hybrid catfish and carp were shown by [37, 47] Also, found the same results but for grass carp. MDA concentrations have been shown to be often used as markers of lipid peroxidation [50]. Threonine and/or coated butyric acid supplements considerably reduced the MDA value. Accordingly, the present research indicated that coated butyric acid and/or threonine reduce oxidative stress and boost fish's antioxidant capacity.By strengthening the activity of antioxidant enzymes like CAT and SOD and lowering the concentration of MDA, especially in G6, after concurrently administering threonine and butyric acid, a synergistic impact in antioxidant enzyme activity was noted.Similar results were reported by [51] in juvenile grass carp. The supplementation of L-threonine and sodium butyrate also led to lower activity of aspartate aminotransferase and alanine aminotransferase.

AST and ALT activity lowered significantly for a fish supplemented with 0.48 g L-threonine and 0.3 g sodium butyrate in its diet. ALT and AST are established and sensitive biomarkers for identifying hepatocyte injury and liver necrosis [52]. In the present study, the activity levels of ALT and AST exhibited a significant reduction compared to the control group. As a result, no indications of inflammation were detected in the liver of *O. niloticus*, suggesting that there was no hepatic injury or damage. Our findings align with [53], who found the same result for blunt snout bream, and [54] for grass carp.

Our study indicated that dietary L-threonine and sodium butyrate supplementation diets enhanced digestion and digestibility for Nile tilapia, which was reflected by a significant increase in lipase and amylase compared with the control group.

Total protein demonstrated a non-significant increase for the treated groups compared to the control in the 3rd week. But at the end of the experiment, it showed a significant increase, especially in the supplemented groups 4, 5, and 6 (5.07, 5.85, and 7.49, respectively). Also, the albumin level was significantly higher in treated groups at the half and ends of the experiment. The current findings agreed with those reported by [39]. Data from the current study shows that the nonspecific immune response, especially the nitric oxide level, was significantly higher for the supplemented groups compared with the control one. That might be because of the ability of these dietary additives to improve the immune system activity. Supplemented groups increased the IgM significantly higher than the control group. The highest level was for the mixed group, as IgM is the primary defense molecule that mediates the immune response in freshwater fish [55–57].

In our study, there were significant improvements in intestinal villi height and their thickness. Meanwhile, an increase in surface area is capable of greater absorption of digested nutrients [58]. The data above suggested that diet-elevated threonine increases the absorptive capacity of Nile tilapia, as noticed by [54] in sub-adult grass carp and [45] in juvenile carp; in terms of intestinal morphology, the supplementation of L-threonine and sodium butyrate resulted in improvements in intestinal villi height, thickness, and surface area. This suggests an increased absorptive capacity for digested nutrients [59]. Similarly, threonine deficiency, on the other hand, reduced juvenile grass carp's intestinal function and morphology, as suggested by [60].

## Conclusion

Based on our findings, the recommended dose for Nile tilapia fingerlings was encapsulated butyric acid 0.3 g/kg combined with L-threonine 0.48 g/kg diet, which remarkably enhances growth performance parameters, immune response, antioxidant status, intestinal villi length, and net profit. Future research to evaluate the advantages of a dietary combination of encapsulated butyric acid and L-threonine at varying doses in aquaculture is encouraged by the findings of this investigation. However, additional research is required to ascertain these compounds' potential impact on other fish species.

## Data Availability

The data presented in this study are available within the article.

## Abbreviations

ALT: Alanine transaminase
AST: Aspartate aminotransferase
ButiPEARL: Encapsulated form of butyric acid
CAT: Catalase
FCR: Feed conversion ratio
HIS: Hepatosomatic index
IgM: Immunoglobulin M
MDA: Malondialdehyde
MSS: Methanesulfonate
NP: Net profit
PBS: Phosphate-buffered saline
SCFA: Short-chain fatty acid
SEM: Scanning electron microscopy
SGR: Specific growth rate
SOD: Superoxide dismutase
TC: Total cost
TFC: Total fixed cost
TR: Total return
TVC: Total variable cost
WG: Weight gain
WGR: Weight gain rate
